# The Comparison of Physical Activity, Sedentary Behavior, and Mental Health between Early Menopausal Women and Age-Matched General Middle-Aged Women

**DOI:** 10.3390/ijerph18147256

**Published:** 2021-07-07

**Authors:** Ji-Su Kim, Ju-Pil Choe, Jeong-Hui Park, Eunhye Yoo, Jung-Min Lee

**Affiliations:** 1 Graduate School of Physical Education, Kyung Hee University (Global Campus), 1732 Deokyoungdaero, Gi-heung-gu, Yongin-si 17014, Korea; khutkdkimjisu@khu.ac.kr (J.-S.K.); jupilchoe@khu.ac.kr (J.-P.C.); jeonghee@khu.ac.kr (J.-H.P.); 2 Department of Physical Education, Seoul National University, 1 Gwanakro, Gwanakgu, Seoul 08826, Korea; yeh04@snu.ac.kr; 3 Sports Science Research Center, Kyung Hee University (Global Campus), 1732 Deokyoungdaero, Giheung-gu, Yongin-si 17014, Korea

**Keywords:** physical activity, sedentary behavior, mental health, early menopause, middle-aged women

## Abstract

The current study is to examine the differences in physical activity (PA), sedentary behavior (SB), and mental health (i.e., stress, depression, and suicidal behaviors) between early menopausal women and age-matched general middle-aged women. Among 1348 participants in South Korea, 674 participants who experienced menopause before the age of 45 were defined as the early menopausal group, and 674 women who experienced menopause from 45 years to 55 years were classified as the general group by matching age based on early menopausal women. PA, SB, and mental health were evaluated by using the Global Physical Activity Questionnaire (GPAQ). An independent t-test was used to compare the associations of PA, SB, and mental health between the two groups. To demonstrate the predictors of early menopause, variables in the study were analyzed by multinomial logistic regression. The main findings were that moderate-to-vigorous PA (MVPA) and light PA (LPA) had significant differences between the two groups, but SB had no significant differences. In mental health, only perceived stress had significant differences in this study. The moderate level of stress in the early menopausal group was twice as high as that of the general group, and the severe level of stress was even 2.6 times higher than the general group. PA plays an essential role in mitigating the causes of mortality and the risk of various chronic diseases and improving quality of life; thus, the main findings of this study could be important to provide insights on the corresponding impact between early menopausal women and PA to encourage their healthy lifestyle. Further longitudinal studies are needed to examine the mechanisms underlying the effects of PA, SB, and mental health on early menopausal women.

## 1. Introduction

As life expectancy increases, the importance of health care for individuals aged 40 to 60 years old is increasingly emphasized in modern society to enhance the quality of life in old age. However, middle-aged women have difficulty maintaining well-being because they may experience menopause syndrome due to ovarian weakness and hormonal deficiency [1], and it also leads to increased risk of chronic diseases such as cardiovascular diseases, stroke, and osteoporosis [2].

Menopause is a permanent cessation of menstruation resulting from the loss of ovarian follicular function that occurs in the woman’s midlife [3]. It is difficult to indicate the definite onset age of menopause, but community-based studies suggest that the distribution of menopausal age represents a normal distribution that ranges from age of 40 to end around the age of 54 and typically clustering around the ages 45–55 [4,5,6]. Additionally, one previous study reports that the average age of menopause is 54 in Europe, 51.4 in North America, 48.6 in Latin America, and 51.1 in Asia [7]. According to these statistics, the age of 45–55 is considered as a common natural menopausal age, and before the age of 45 refers to early menopause [8].

In the menopausal transition period, women may experience some physical/physiological changes in their body as a result of hormonal changes, including hot flashes, insomnia, irritability, mood swings, depression, and anxiety disorders [9,10]. In particular, early menopause has been announced to be related to all-cause mortality [11,12] and has a lot of negative effects on cardiovascular disease [13], neurological diseases, and osteoporosis [14]. According to the World Health Organization (WHO) Guidelines, middle-aged adults should participate in at least 150 min of moderate-intensity PA (activity types between 3 and 6 metabolic equivalents (METs) per week, or at least 75 min of vigorous-intensity PA (activity types 6 METs) per week, or a proportionate combining of moderate-to-vigorous intensity PA for their well-being [15]. WHO (2020) stated that all middle-aged adults need high-intensity or moderate-intensity exercise activities associated with all major muscles more than two days a week [15]. However, previous studies have shown that daily energy expenditure decreases significantly in middle-aged women and an alteration toward a more sedentary lifestyle may deteriorate their health and the quality of life during the menopausal transition [16,17].

Based on the results of precedent studies, early menopausal women have a high risk of all-cause mortality [11,12], chronic diseases (i.e., heart disease, ischemic stroke) [13,14], osteoporosis, and fracture [18,19]; these early menopausal women tended to be less active and spend more time on sedentary behavior (SB) than ordinary people. Unfortunately, the longer SB becomes, the more stress or depression one feels in everyday life, and the negative effects of life during early menopause have been confirmed in several studies [20,21,22]. Several prior studies supported the fact that the quality of life of women who enter into menopausal transition at earlier ages may be more adversely affected than general middle-aged women [23,24]. Although many previous studies have revealed the positive effect of PA on menopausal women [25,26,27], there are few comparisons of PA, SB, and mental health between early menopausal women and general middle-aged women.

Therefore, the purpose of this study is to systematically investigate the association between PA, SB, and mental health in early menopausal women, compared to the age-matched general middle-aged women.

## 2. Method

### 2.1. Design

The Korea National Health and Nutrition Examination Survey (KNHANES) is a population-based, cross-sectional survey designed to evaluate the health-related behavior, health status (i.e., mental health), and nutritional status by using the Global Physical Activity Questionnaire (GPAQ), controlled by the Korea Centers for Disease Control and Prevention (KCDCP). The questionnaire used a Korean-language version of the GPAQ, which was proven reliable and valid [28]. The variables that PA, SB, and perceived stress were assessed in middle-aged women who agreed to participate in the seventh year (2016–2018) of the KNHANES. This study used the data of KNHANES that was approved by the Institutional Review Board of the Korea Centers for Disease Control and Prevention (2015-01-02-6C).

### 2.2. Study Participants

A total of 24,269 Korean women responded to the 2016–2018 KNHANES survey, with 8150 in 2016, 8127 in 2017, and 7992 in 2018. Among those, women who responded that they were non-menopausal (*n* = 19,377) were excluded (*n* = 4892). Additionally, those with missing information about weight, height, PA, SB, and participation rate of aerobic activity were excluded (*n* = 4218); thus, 674 early menopausal women in whom menopause had occurred before the age of 45 remained. Furthermore, the participants’ anthropometrics information was calculated as mean ± SD. Therefore, the 1348 participants of the current study consisted of 647 participants of the early menopausal group and 647 participants of the age-matched general middle-aged group, based on the onset age of menopause before the age of 45 and age ranging from 45 to 55, respectively.

### 2.3. Measures

#### 2.3.1. Physical Activity (PA)

Among the questions of GPAQ, the daily physical activity was evaluated through the following questions: “How many days do you usually do moderate-to-vigorous intensity physical activity for a week?” and “How many hours do you usually do moderate-to-vigorous intensity physical activity for a week?” PA was calculated by “MET level × minutes × number of activities per week” for each intensity, with 6.0 METs for vigorous physical activities, 3.0 METs for moderate physical activities, and 2.0 METs for light physical activities [29]. Additionally, we set the walking variable as a light PA for comparing the level of participation in light physical activity. The walking variable included the following questions: “How many days have you walked at least 10 min at a time during the latest week?” and “How long are you usually walking in a day?” Further, a 8-point scale (i.e., 1 = never, 2 = 1 day, 3 = 2 days, 4 = 3 days, 5 = 4 days, 6 = 5 days, 7 = 6 days, 8 = every day) was added to the former question. The participation rate of the aerobic activity was measured by asking, “Did you participate in 150 min or more of moderate physical activity, or 75 min or more of vigorous physical activity, or mixed moderate and vigorous physical activity (i.e., vigorous 1 min = moderate 2 min) for a week?” A 2-point scale (i.e., 0 = never, 1 = participation in aerobic exercise) was added to this question.

#### 2.3.2. Sedentary Behavior (SB)

SB was calculated by “MET level × minutes × number of activities per week” with 1 METs [29]. The SB variable was assessed by asking the question “How many hours do you usually sit or lie down in a day?” The question for SB was calculated as the amount of time by sitting or lying down at home, at work, when moving around, or hanging out with friends, excluding time in sleep (i.e., sitting on a desk, sitting with friends, moving by car, bus, or train, reading a book, writing, playing a card game, watching TV, playing a video/computer game, using the internet, and listening to music).

#### 2.3.3. Stress

The perception of stress was assessed through the following question: “How much stress do you usually feel in your daily life?” A 4-point Likert Scale (i.e., 1 = severe, 2 = moderate, 3 = mild, 4 = very mild) was used for the perceived stress question.

#### 2.3.4. Depression

Depression was investigated through the following questions: “Have you ever felt sad or desperate, which interferes with daily life for 2 consecutive weeks or more in the past year?” and “Have you ever taken counseling for a mental problem (i.e., through the visit, telephone, internet) in the past year?” The responses were also measured as “yes” or “no” in both questions.

#### 2.3.5. Suicidal Behaviors

Suicidal behaviors were examined by asking questions on suicide thoughts, suicide plans, and suicide attempts. The thought of suicide asked, “Have you ever seriously thought of attempting suicide in the past year?” The suicide plan asked, “Have you ever made any specific plans to attempt suicide in the past year?”, and the suicide attempt asked, “Have you ever actually attempted suicide in the past year?” The responses were also measured as “yes” or “no” in all questions.

### 2.4. Data Analysis

The participants’ characteristics and anthropometric information were summarized by SPSS 25.0 version (SPSS Inc., Chicago, IL, USA). The participants’ personal information (i.e., age, education, average monthly income, and occupation) was examined by descriptive statistics and anthropometrics information (i.e., height, weight, and BMI) were also analyzed by an independent t-test for continuous variables. The independent t-test was utilized to investigate anthropometrics’ differences between early menopausal and the general group, and a *p*-value was used to evaluate whether there are significant differences between the two groups. Additionally, to demonstrate the predictors of early menopause, some variables (i.e., PA, SB, participating in aerobic PA, stress, depression, and suicidal behaviors) in the present study were examined by multinomial logistic regression analysis, and results were presented as odds ratios (OR) with 95% confidence intervals (95% CI) and statistical significance set by *p* < 0.05.

## 3. Results

Table 1 summarizes the participants’ demographic information (i.e., age, education, average monthly income, and occupation of participants) of middle-aged women, comparing between the early menopausal group (n = 674) and general group (n = 674), by using descriptive statistics. Generally, the number of participants in the age of over 70 was relatively higher, and the education level of the menopausal group was the highest, with 55.78% in elementary school graduates, followed by 20.17% in high school graduates. Likewise, that of the general group were elementary school graduates with 43.02%, followed by high school graduates with 25.37%. Additionally, more than half of the participants earned less than KRW 2000 thousand (USD 1798), and the occupation of participants presented the highest portion in unemployment, followed by laborers in both groups. The early menopausal group indicated 153.67 ± 6.45 cm for height, 57.72 ± 9.73 kg for weight, and 24.43 ± 3.52 kg·m^−2^ for BMI; similarly, the general group showed 155.54 ± 5.87 cm for height, 57.16 ± 8.15 kg for weight, and 23.93 ± 3.20 kg·m^−2^ for BMI. The results indicated that there were no significant differences in height, weight, and BMI between the two groups (*p* > 0.05).

Table 2 is the results of multinomial logistic regression of factors in stress and participating in aerobic PA that can predict the association of participating in PA, SB, and mental health in early menopausal women, compared to general women. According to Table 2, the moderate-to-vigorous physical activity (MVPA) (OR = 0.998; CI = 0.998–0.998; *p* < 0.001) and the light physical activity (LPA) (OR = 0.996; CI = 0.995–0.997; *p* < 0.001) measured by questionnaires had significant differences between the two groups, and MVPA and LPA in the early menopausal group tended to decrease rather than in the general group. However, the result of SB in this study had no significant differences between the two groups (OR = 1.000; CI = 1.000–1.001; *p* > 0.05). Specifically, in the rate of participating in aerobic PA, there were significant differences between the two groups—lower participation in aerobic PA was observed in the early menopausal group (OR = 2.827; CI = 2.109–3.789; *p* < 0.001), compared to the general group. The result of participating in aerobic PA demonstrated that women with early menopause are 2.8 times more likely than general women to not participate in aerobic PA. In addition, early menopausal women are more likely to feel stress than general women (*p* < 0.001), with more than 2 times in moderate-level of stress (OR = 2.006; CI = 1.367–2.945; *p* < 0.001) and 2.6 times in severe level of stress (OR = 2.635; CI = 1.314–5.282; *p* < 0.01). Although there were no significant differences in depression and suicidal behavior between the two groups, given that the odds ratio of suicidal behavior, suicidal behavior is 1.5 times more likely in menopausal women than in general women (OR = 1.572; CI = 0.774–3.191; *p* = 0.211).

**Figure 1:**
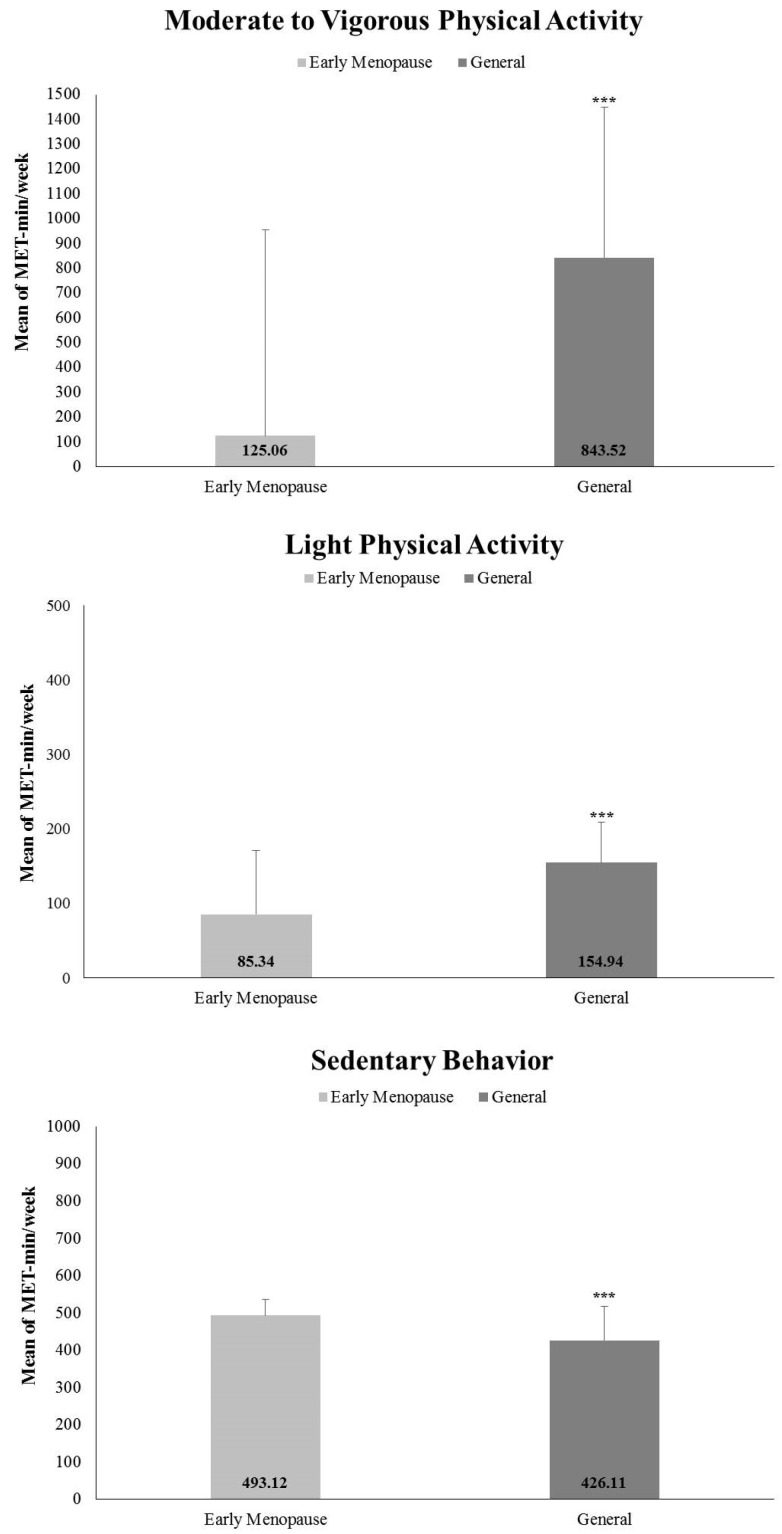
Comparison of average physical activity time and sedentary behavior during a week between early menopausal and general group. The significant differences between the two groups; *** *p* < 0.001, error bars represent the 95% confidence interval.

Figure 1 illustrates the comparison of average PA time and SB during a week between the early menopausal and general groups. The average participation in MVPA and LPA was 125.06 ± 438.36 METs-minute per week and 42.67 ± 55.71 METs-minute per week in the early menopausal group, but the participants in the general group were 843.52 ± 1409.76 METs-minute per week and 77.47 ± 89.23 METs-minute per week, respectively. The results revealed significant differences in MVPA (*p* = 0.000; 95% CI = −830.01–−606.90) and LPA (*p* = 0.000; 95% CI = −42.75–−26.85) measured by questionnaire between the menopausal and general groups. Furthermore, the average time of SB also showed a significant difference between the two groups (*p* = 0.000; 95% CI = 43.54–90.49), with 493.12 ± 223.73 METs-minute per week in the early menopausal group and 426.11 ± 215.53 METs-minute per week in the general group.

## 4. Discussion

Middle-aged women who experience menopause earlier are more exposed to risks of various health-related problems [12,14,18,19]. Several previous studies have already revealed the positive role of PA in menopause [25,26,27], but there is still a lack of studies investigate the associations of PA, SB, and mental health in early menopausal women. Despite PA being a crucial variable to predict a high risk of mortality and diseases in early menopausal women [30,31], there are some inconsistent results that PA is not related to the risk of early menopause [32,33]. Therefore, the purpose of this study is to systematically investigate the association between PA, SB, and mental health in early menopausal women, compared to the age-matched general middle-aged women.

Our study provides novel findings that MVPA and LPA had significant differences between the two groups, and both MVPA and LPA results indicated that those women in the early menopausal group were less likely to participate in PA than the general group (*p* < 0.001). In addition, there were also significant differences in the rate of participating in aerobic PA between the two groups, and the present study demonstrated that women with early menopause are 2.8 times more likely not to participate in aerobic PA than the general women (*p* < 0.001, OR = 2.827). Early menopausal women have a high risk of all-cause mortality, chronic diseases (i.e., heart diseases, ischemic stroke), osteoporosis, and fracture, and these poor health statuses may derive from less participation in regular PA [11,12,13,14,18,19].

However, there were no significant differences between the two groups (*p* > 0.05) in SB because the majority of participants in this study were elderly women in both groups. Although the increased time of SB leads to low energy expenditure and can be a public health risk factor, the average spent time of SB in older adults is equating to 65% to 80% of their waking hours [34]. Prolonged SB in the middle-aged is associated with several sociodemographic (i.e., older age, higher BMI), behavioral (i.e., lower MVPA), environmental (i.e., region of residence in non-stroke belt/buckle), and seasonal (winter season) factors [35]. In particular, old-aged women are more likely to increase time spent in SB because of their higher rate of retirement, compared to younger women [36]. Even though there were no significant differences in SB between the two groups, prolonged SB in elderly women is highly related to less PA and risk of chronic diseases [36]; therefore, future studies require a systematic investigation of SB by age.

Additionally, the current study found that the association between PA and perceived stress showed significant differences between the two groups (*p* < 0.001). The moderate level of stress in the early menopausal group was more than twice as high as that of the general group, and the severe level of stress was even 2.6 times higher than the general group. The finding is consistent with other studies’ results. A review study by Stults-Kolehmainen et al. revealed that many studies examining older adults were more likely to show an inverse association between PA and perceived stress [37]. Another previous study also found a statistically significant positive effect of stress on PA [38]. As a result, it can be assumed that perceived stress is highly associated with the level of PA. The weak health status of early menopausal women (i.e., high risk of all-cause mortality, chronic diseases, osteoporosis, and fracture) may derive from less participation in PA. To some extent, less participation in PA can also be related to increased stress in early menopausal women. Although there were no significant differences in depression and suicidal behaviors between the two groups, in the present study, the early menopausal group was 1.5 times more likely to attempt suicidal behaviors than the general group. In fact, increased stress can lead people to depression and suicidal behaviors [39,40]. Thus, we suggest that recommendation for inducing PA participation is needed to reduce stress and prevent the risk of depression and suicide in early menopausal women.

The current study revealed several positive strengths. First of all, this study is the first to investigate PA and mental health based on age-matched, middle-aged women comparing the onset age of menopause, divided into the early menopausal group and general group. Moreover, this study is the first to examine the association of PA, SB, and mental health, related to the onset age of menopause, and revealed significant differences between the early menopausal and general groups. However, there are also a few limitations in this study. Since the study utilized data obtained from the self-reported questionnaire in South Korea, which can be underestimated or overestimated by individuals [41], it may less reliable, compared to the objective measurement. Therefore, using valid measurement tools is needed for obtaining more accurate data on PA in future studies. In addition, several factors induce early menopause such as genetic factors, irregular menstrual cycle, smoking, drinking, and some medical treatments (i.e., surgery or chemotherapy) [42], but the current study was unable to investigate the specific causes of early menopause for individuals in the early menopausal group. Thus, further studies need to scrutinize more lines of evidence of differences in various causes of early menopause from the same perspective. Although the fact that significant correlation between PA and depression has been verified by several studies [43,44,45], this study has limitations in finding differences in depression between the early menopausal and general groups, which showed significant differences in PA. Other studies in the future will need to demonstrate more lines of evidence with various countries in the world regarding the association of PA, SB, and mental health between early menopausal and general women.

## 5. Conclusions

In conclusion, the study revealed significant differences in PA and perceived stress between early menopausal and general women and found a close association between physical inactivity and more stress in early menopausal women. Therefore, the main findings of this study could be important to provide insights on the corresponding impact between early menopausal women and PA to encourage their healthy lifestyle. Further longitudinal studies are needed to examine the mechanisms underlying the effects of PA, SB, and mental health on early menopausal women.

## Figures and Tables

**Table 1:** Participants’ characteristics information between the early menopausal group and the general group.

Variable		Early Menopause Group (*n* = 674)		General Group (*n* = 674)	
		No. (%)	Mean ± SD	No. (%)	Mean ± SD
Age (year)	30–39	5 (0.74)	37.60 ± 1.14	5 (0.74)	37.60 ± 1.14
	40–49	77 (11.42)	45.79 ± 2.55	77 (11.42)	45.79 ± 2.55
	50–59	143 (21.22)	55.66 ± 2.79	143 (21.22)	55.66 ± 2.79
	60–69	169 (25.07)	64.89 ± 3.00	169 (25.07)	64.89 ± 3.00
	>70	280 (41.54)	76.09 ± 3.46	280 (41.54)	76.09 ± 3.46
Anthropometrics	Height (cm)		153.67 ± 6.45		155.54 ± 5.87
	Weight (kg)		57.72 ± 9.73		57.16 ± 8.15
	BMI (kg·m^−2^)		24.43 ± 3.52		23.93 ± 3.20
Education	<Elementary School	376 (55.78)		290 (43.02)	
	<Middle School	84 (12.46)		102 (15.13)	
	<High School	136 (20.17)		171 (25.37)	
	>Undergraduate	77 (11.42)		111 (16.46)	
Average Monthly Income	<1000 thousand KRW	168 (24.92)		124 (18.39)	
	1000–2000 thousand KRW	178 (26.40)		166 (24.62)	
	2000–3000 thousand KRW	168 (24.92)		183 (27.15)	
	>3000 thousand KRW	159 (23.59)		198 (29.37)	
Occupation	Administrators, managers, and professionals	25 (3.70)		26 (3.85)	
	Office workers	27 (4.00)		18 (2.67)	
	Service workers and shop sales workers	83 (12.31)		62 (9.19)	
	Skilled agricultural and fishery workers	43 (6.37)		29 (4.32)	
	Machine operators and assemblers	25 (3.70)		13 (1.93)	
	Laborers (not elsewhere classified)	93 (13.79)		96 (14.29)	
	Jobless (e.g., housewife, students)	378 (56.08)		430 (63.8)	

SD: standard deviation; BMI: body composition index. The total number of persons responded 670 at height and BMI in the early menopausal group and 672 at height and BMI in the general group, 0.6% and 0.3% missing from the table, respectively (did not answer); there are no significant differences in height, weight, and BMI between early menopausal and general group (*p* > 0.05); Education: educational status (for example, “elementary school” means those graduated up to elementary school), USD 1 = KRW 1124.5 (Korean Won, 14 April 2021).

**Table 2:** Multinomial logistic regression of factors in stress and participating in aerobic physical activity.

Variable		Early Menopause Group (*n* = 674)						
		*b*	S.E	*Wald*	*p*	*Exp(b)*	*Exp(b)* 95% Confidence Intervals	
							Lower	Upper
Physical Activity	MVPA	−0.002	0.000	139.779	0.000 ***	0.998	0.998	0.998
	LPA	−0.004	0.001	44.413	0.000 ***	0.996	0.995	0.997
	SB	0.000	0.000	1.808	0.179	1.000	1.000	1.001
Participating in Aerobic PA	Yes	0	.	.	.	1.000	.	.
	No	1.039	0.149	48.343	0.000 ***	2.827	2.109	3.789
Stress	Very Mild	0	.	.	.	1.000	.	.
	Mild	0.401	0.153	6.866	0.009 **	1.493	1.106	2.015
	Moderate	0.696	0.196	12.650	0.000 ***	2.006	1.367	2.945
	Severe	0.969	0.355	7.454	0.006 **	2.635	1.314	5.282
Depression	No	0	.	.	.	1.000	.	.
	Yes	−0.150	0.279	0.291	0.590	0.860	0.498	1.487
Suicidal Behavior	No	0	.	.	.	1.000	.	.
	Yes	0.452	0.361	1.566	0.211	1.572	0.774	3.191

Reference group: general group; *** *p* < 0.001, ** *p* < 0.01, OR: odds ratio, MVPA: moderate-to-vigorous physical activity, LPA: light physical activity, SB: sedentary behavior; participating in aerobic PA (no): this means not participating in PA (vigorous-intensity PA 1 min corresponds to moderate-intensity PA 2 min) or vigorous-intensity PA for two and a half hours or more per week, or vigorous-intensity PA for an hour and a quarter.

## Data Availability

The datasets used and/or analyzed during the current study are available from the corresponding author on reasonable request.
